# A comparison of the transillumination-assisted technique versus midline approach technique in novices: a prospective randomized controlled trial about the Bonfils intubation fiberscope

**DOI:** 10.1186/s12871-017-0322-6

**Published:** 2017-02-21

**Authors:** Jian Wang, Lan Yuan, Guoqiang Fu, Wei Tang, Guijie Yu, Feng Guo, Jiangang Song

**Affiliations:** Department of Anesthesiology, Shanghai Shuguang Hospital, Shanghai University of Traditional Chinese Medicine, Shanghai, 201203 China

**Keywords:** Bonfils intubation fiberscope, Tracheal intubation, Transillumination-assisted technique

## Abstract

**Background:**

The present study aimed to compare the safety and efficacy for novices to conduct intubation with the Bonfils intubation fiberscope (BIF) using the transillumination-assisted or midline approach technique in patients with normal airways.

**Methods:**

In this prospective randomized control study, 10 trainees were assigned to the transillumination-assisted technique group (T group) or the midline approach technique group (R group). Each trainee was required to conduct intubation in 50 patients. The primary outcome was intubation time. The secondary outcomes were success rate (%), number of attempts, and complications.

**Results:**

Among the cases of successful intubation, the intubation time was not significantly different between the two groups (*P* > 0.05). The overall success rate of intubation was not significantly different between the two groups (*P* > 0.05). The intubation success rates at the first, second, and third attempts as well as the average intubation times were similar between the two groups (*P* > 0.05), but in patients receiving successful intubation at the second attempt, the intubation time was longer in the T group (*P* = 0.0006). The incidences of dry throat, sore throat, and hoarseness were higher in the T group (all *P* < 0.05).

**Conclusions:**

For patients with a normal airway, the transillumination-assisted technique was unlikely to increase the success rate of intubation with the BIF compared with the midline approach technique, but led to more complications.

**Trial registration:**

ChiCTR-INR-16009967, retrospectively registered on November 22, 2016

**Electronic supplementary material:**

The online version of this article (doi:10.1186/s12871-017-0322-6) contains supplementary material, which is available to authorized users.

## Background

Establishing a successful endotracheal intubation is important for pulmonary ventilation and gas exchange during anesthesia and critical care. Failed or difficult endotracheal intubation is an important cause of morbidity and mortality during anesthesia [1]. A variety of methods and tools (such as direct laryngoscope, video laryngoscopes, and fiber optic bronchoscope (FOB)) have been used for endotracheal intubation, but no single device or method can be applied to all airway scenarios. Therefore, anesthesiologists have to master a variety of airway devices.

Among these devices, the Bonfils intubation fiberscope (BIF) is widely used. The BIF is a rigid, long, slender device with a curved tip. A tracheal tube is loaded onto the shaft of the BIF, inserted into the mouth, and advanced to the glottis aperture. After identifying the vocal cords, the tracheal tube is placed into the trachea [2, 3]. The BIF can be used for the endotracheal intubation normal airways, but is especially useful for difficult tracheal intubations for patients with limited cervical mobility or limited mouth opening [4, 5]. The most important disadvantage of the BIF is the long learning curve [2, 6].

Two methods are commonly used for targeting the glottis using BIF: the midline approach technique and the transillumination-assisted technique. The midline approach technique is one of the most common techniques for BIF; the operators directly observe through the eyepieces to look for the glottis under direct vision [6], intra-oral mist, secretions, or contamination impair the vision during intubation and are the main reasons for BIF intubation failure. Recent studies showed that the application of the transillumination-assisted technique can improve the success rate of intubation [7]. In the transillumination-assisted technique, an external light is used to locate the glottis through the cricothyroid membrane [8]. Since the transillumination-assisted technique does not entirely rely on the direct observation of the glottis through the eyepieces, it is less likely to be affected by blurred vision.

Nevertheless, which technique is easier for trainees remain to be shown. Therefore, we hypothesized that the transillumination-assisted technique have a higher success rate for intubations performed by trainees. Therefore, this study compared the transillumination-assisted and midline approach techniques in terms of intubation time, success rate, and intubation-related complications, hence providing clinical data to further optimize the learning and training process of BIF-guided tracheal intubation.

## Methods

### Study design

This prospective randomized controlled trial was conducted at the Anesthesiology Department of Shuguang Hospital Affiliated to Shanghai University of Traditional Chinese Medicine, the study was approved by the ethics committee of the hospital. The trial is registered with Clinicaltrials.gov (ChiCTR-INR-16009967), available online: http://www.chictr.org.cn/showproj.aspx?proj=15963.

Between January 2013 and June 2014, 500 patients undergoing tracheal intubation were recruited. Tracheal intubation operations were conducted by 10 students (trainees) from the Anesthesiology Department of Shuguang Hospital Affiliated to Shanghai University of Traditional Chinese Medicine. These trainees had ≤2 years of doctor’s qualification, but did not have experience with the BIF. They were assigned to the transillumination-assisted technique group (T group) or the midline approach technique group (R group) using a simple randomization method based on a random number table.

The trainees were provided standard training by a chief physician who was considered as the observer and was skilled in using the Bonfils optical stylet (>100 successful unsupervised intubation operations). The training included explaining the theory of the BIF technique, watching teaching videos, and guiding the trainees to conduct tracheal intubation using the BIF in manikins (Shanghai Xinman Co., Ltd., Shanghai, China) [9]. After the trainees had successfully conducted tracheal intubation in manikins for three times, they were randomized and performed intubation operations.

### Patients

The inclusion criteria were: 1) patients undergoing elective surgery under general anesthesia; 2) age 18–65 years; 3) ASA grade I or II; 4) Mallampati grade I–II; 5) body mass index (BMI) of 18–25 kg/m^2^; and 6) signed the written informed consent. The exclusion criteria were: 1) severe cardiopulmonary system diseases; 2) coagulation disorders; 3) missing teeth or removable denture; 4) obesity; or 5) difficult airway.

### Blinding

Since this test could not be blind to observers and operators, only the patients were blinded to the kind of intubation.

### Preparation prior to intubation

The BIF stylet (Karl Storz, Tuttlingen, Germany) was connected to the light source, the objective lens was cleaned with alcohol, and the focal length of the eyepiece was adjusted. The shaft was lubricated with paraffin oil and nested with a suitable type of tracheal tube whose front end was 0.5 cm beyond the front end of the lens shaft; 7.5- and 7-mm tracheal tubes were used for male and female patients, respectively.

Prior to operation, the patients were fasted for 8 h and did not receive any medication. In the operation room, the patient was placed in a supine position, and the venous pathway was opened. Blood pressure, heart rate, electrocardiogram, oxygen saturation, and train-of-four stimulation (TOF) were routinely monitored. The bispectral index (BIS) was monitored using an ASPECT 2000 monitor (Aspect A-2000, MA, USA). The patient was given preoxygenation at 8–10 L/min for 3 min, followed by anesthetic induction with an intravenous injection of 0.05 mg/kg of midazolam, 2 μg/kg of fentanyl, 2 mg/kg of propofol, and 0.6 mg/kg of rocuronium, as well as tracheal intubation after the BIS value was 40–60 and TOF indicated the disappearance of T2.

### Tracheal intubation with transillumination-assisted technique

**Fig. 1:**
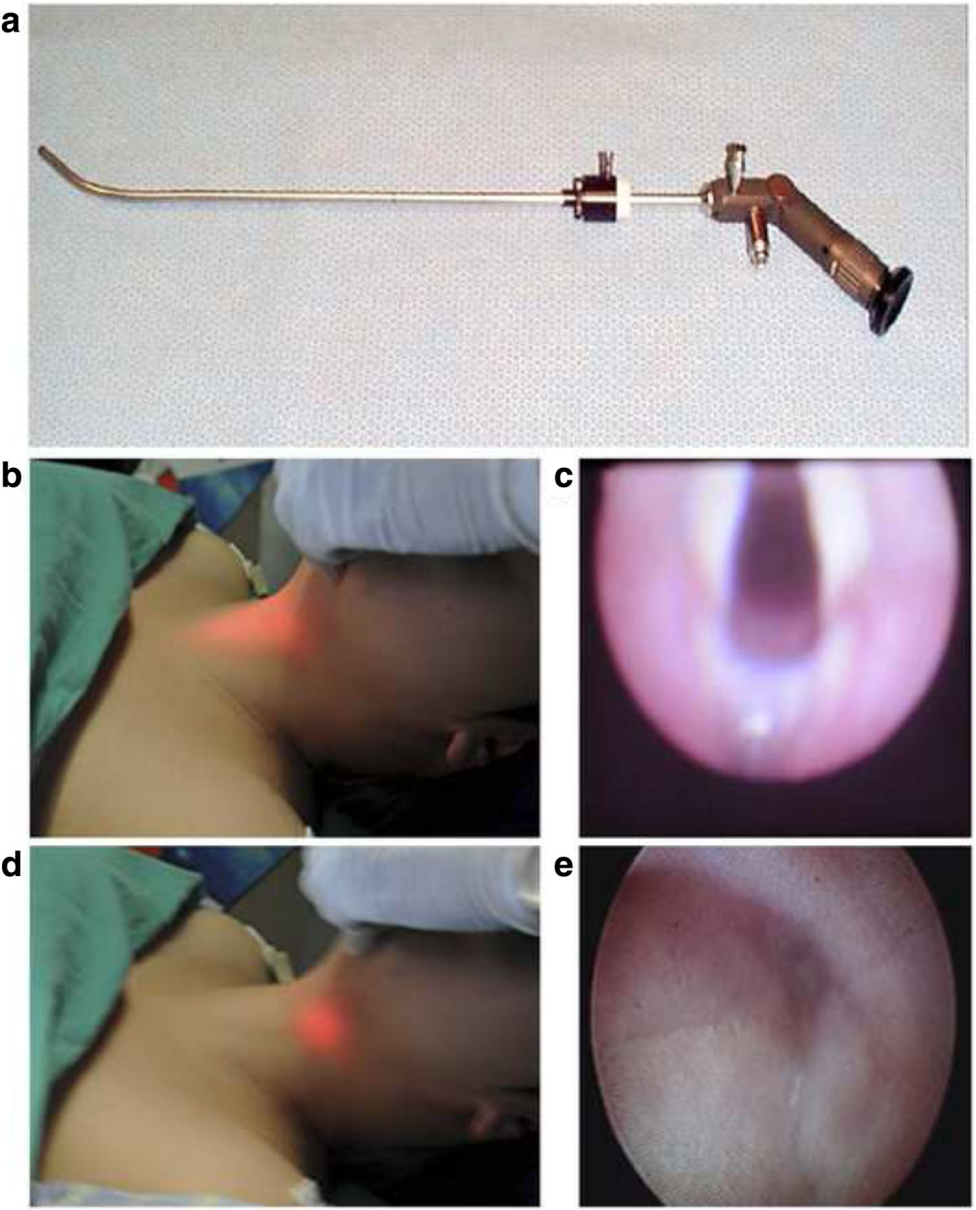
Transillumination-assisted technique and midline approach technique. *Note*: In intubation using the transillumination-assisted technique, the Bonfils optical stylet (**a**) with a preset tracheal tube was slowly inserted into the upper respiratory tract along the midline. If a centered bright spot was visible in the cricothyroid membrane region of the neck (**b**), it might indicate that the laryngoscope tip was located near the glottis, where the vocal cords, annulus tracheae, and other structures were visible (**c**). If the laryngoscope tip was deviated to one side, the spot was more focal and darker (**d**), where sinus piriformis, glossoepiglottic folds, and other structures were visible (**e**)

The head of the patient was maintained in a neutral position without flexion and extension movements. The operator stood near the left side of the patient’s head. Then, the operator lifted the jaw of the patient with his left hand and inserted the optical laryngoscope into the mouth along the incisor midline with the right hand. The operator would look for the brightest transilluminated spot to identify the glottis (Fig. 1). The stylet position was identified under the eyepiece: if it was located at the superior glottis or entered the trachea, the tracheal tube was inserted into the trachea along the lens shaft. However, if the glottis or tracheae ring was invisible, the optical stylet position was adjusted again to continuously look for the brightest spot at the neck midline, and the aforementioned procedures were repeated [7] (Fig. 1).

### Tracheal intubation via midline approach technique

The operator directly observed through the eyepieces to adjust the depth and the direction of the lens body, thereby to look for the glottis and insert the tracheal tube into the trachea along the lens body under direct vision. After successful tracheal intubation, the optical stylet was retracted along the physiological curvature of the oropharynx.

### Outcomes

The primary outcome was the intubation time. The secondary outcomes were success rate, number of attempts, and complications.

All intubation operations were observed by the same observer, who was in charge of recording experimental results, to avoid interobserver bias. This observer also recorded the characteristics of the patients, information on operators, and complications. The data included intubation attempts, success rate of first intubation, overall success rate of three attempts of intubation, intubation time, and intubation-related complications.

The tracheal intubation time was defined as the time elapsed from the insertion of the front end of the laryngoscope into the mouth to the retraction of the laryngoscope after tracheal intubation was completed. The total duration for intubating a patient could not be longer than 3 min, and the maximum number of attempts allowed was three. The cases with three unsuccessful attempts or total intubation time >3 min were considered intubation failure. In addition, patients with blood oxygenation falling to <92% before intubation could be completed were considered as failed. In all these cases, intubation was completed by the observer.

### Statistical analysis

In a preliminary study, the average intubation time was 30.0 ± 18.4 s using BIF with the midline approach technique. An intubation time difference of 50% was considered to be clinically significant. According to the calculation formula of sample size for two independent samples $$ N={\left[\raisebox{1ex}{$\left({Z}_{\raisebox{1ex}{$\alpha $}\!\left/ \!\raisebox{-1ex}{$2$}\right.}+{Z}_{\beta}\right)\times \sigma $}\!\left/ \!\raisebox{-1ex}{$\delta $}\right.\right]}^2\times 4\left({Q_1}^{-1}+{Q_2}^{-1}\right) $$, where type I error alpha value of 0.05 and power of 90%. Thus a sample size of about 500 patients is needed.

Statistical analyses were performed using the SPSS 13.0 software (SPSS Inc., IL, USA). Categorical data was expressed as number or percentage. Continuous data was expressed as mean ± standard deviation. The categorical data was analyzed using the chi-square test, and the continuous data was analyzed using the Student *t* test. The linear regression analysis about the intubation time and patient’s treatment order were performed. Two-sided *P*-values <0.05 were considered statistically significant.

## Results

### Subjects characteristics

**Fig. 2:**
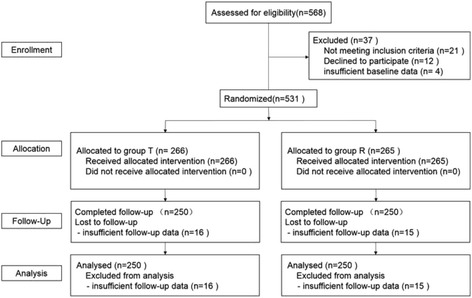
Patient flowchart

A total of 500 ASA grade I or II patients were recruited (Fig. 2). Ten trainees were selected to conduct tracheal intubation (five trainees/group). The general data of the trainees and patients are listed in Table 1. Additional file 1: Figures S1, S2, and S3 present the learning curves of the two groups.

**Table 1:** Characteristics of trainees and airway data of patients undergoing tracheal intubation using the Bonfils optical stylet

	T group (*n* = 250)	R group (*n* = 250)	*P* value
Characteristics of patients			
Gender (male/female)	137/113	133/117	0.720
Age (years)	48.0 ± 12.6	47.1 ± 12.6	0.436
Height (m)	1.68 ± 0.08	1.67 ± 0.08	0.207
Weight (kg)	62.9 ± 9.0	63.1 ± 9.0	0.777
BMI (kg/m^2^)	21.8 ± 2.6	22.2 ± 2.6	0.123
ASA 1/2/3	105/145/0	111/139/0	0.588
Mouth opening < 4 (cm)	0	0	-
Mallampati score (1/2/3)	165/85/0	173/77/0	0.445
Limited neck extension	0	0	-
Thyromental distance <4 cm	0	0	-
Characteristics of trainees			
Number	5	5	-
Age (year)	29.6 ± 1.8	30.2 ± 2.4	0.003
Gender (male/female)	3/2	3/2	-
Doctors’ qualifications (years)	2	2	-

*BMI* body mass index; data were expressed as mean ± standard deviation

### Primary outcome

The number of attempts was similar between the two groups (1.4 ± 0.7 vs. 1.3 ± 0.6, *P* = 0.056). In patients with successful intubation at the second attempt, the intubation time was longer in the T group (67.4 ± 23.7 vs. 52.7 ± 22.1 s, *P* = 0.006), without difference for successful intubation at the first or third attempt (46.7 ± 18.1 vs. 48.6 ± 19.9 s, *P* = 0.348; 80.7 ± 19.8 vs. 63.8 ± 25.0 s, *P* = 0.054). Among all patients with successful intubation, the intubation time was similar between the two groups (52.8 ± 22.2 vs. 49.9 ± 20.7 s, *P* = 0.137). No intubation was failed because of blood oxygenation <92%.

### Secondary outcomes

The success rates of the first, second, and third attempts were similar between the two groups (71.6% vs. 78.0%, *P* = 0.100; 57.8% vs. 65.5%, *P* = 0.301; 58.1% vs. 57.9%, *P* = 0.991). In addition, the overall intubation success rate was not significantly different between the two groups (94.8% vs. 96.8%, *P* = 0.265).

Among patients receiving intubation with the transillumination-assisted technique, intubation was successful at the first, second, and third attempts in 71.6% (179/250), 16.0% (40/250), and 7.2% (18/250) of patients, respectively. Intubation was unsuccessful after three attempts in 5.2% (13/250) of patients, mainly because the trainees failed to find the bright spot. Among patients receiving intubation with the midline approach technique, intubation was successful at the first, second, and third attempts in 78.0% (195/250), 14.4% (36/242), and 4.4% (11/242) of patients, respectively. Intubation was unsuccessful after three attempts in 3.2% (8/250) of patients, mainly because the trainees could not clearly observe the glottis.

Compared to the midline approach technique, the frequencies of dry throat (52.2% vs. 40.4%, *P* = 0.020), sore throat (26.8% vs. 16.8%, *P* = 0.009) and hoarseness (14% vs. 8%, *P* = 0.045) were higher in the transillumination-assisted technique (Table 2).

**Table 2:** Main study results and complications

	T group (*n* = 250)	R group (*n* = 250)	*P* value
Overall intubation success rate (%)	237 (94.8)	242 (96.8)	0.265
Rate of Success at the first attempt (*n*, %)	179 (71.6)	195 (78)	0.100
Succeed at the second attempt (*n*, %)	40 (16.0)	36 (14.4)	0.301
Succeed at the third attempt (*n*, %)	18 (7.2)	11 (4.4)	0.991
Failed after three attempts(n,%)	13 (5.2)	8 (3.2)	0.265
Intubation frequency (*n*, mean ± SD)	352 1.4 ± 0.7	324 1.3 ± 0.6	0.056
Successful intubation time (s)	52.8 ± 22.2	49.9 ± 20.7	0.137
First attempt (s)	46.7 ± 18.1	48.6 ± 19.9	0.348
Second attempt (s)	67.4 ± 23.7	52.7 ± 22.1	0.006^*^
Third attempt (s)	80.7 ± 19.8	63.8 ± 25.0	0.054
Complications			
Dry throat (%)	128 (51.2)	101 (40.4)	0.020^*^
Sore throat (%)	67 (26.8)	42 (16.8)	0.009^*^
Hoarseness (%)	35 (14)	20 (8)	0.045^*^
Trauma (%)	0	0	-

*Note*: For comparison of intubation time and successful intubation time, a difference with *P* less than 0.05 was considered statistically significant, and indicated with “*”. The *P* value was calculated using the independent-sample Student *t* test or chi-square test. Data were expressed as mean ± standard deviation and number (percentage)

## Discussion

This study compared the intubation time, success rate and complications of the BIF conducted by novices using the midline approach technique or the transillumination-assisted technique. It was found that the intubation time and intubation success rate were similar between the two techniques. The intubation time for patients receiving successful intubation at the second attempt was longer with the transillumination-assisted technique than with the midline approach technique. The complication frequency of the transillumination-assisted technique was significantly higher compared to the midline approach technique.

In this study, the overall success rate of intubation with the BIF was 94.8–96.8%. Byhahn et al. reported a success rate of 81.6% in simulating patients with a difficult airway who received tracheal intubation with the BIF [10]. Piepho et al. conducted a study of novices using the BIF in a simulated difficult airway and showed that the success rate of the BIF intubation was 82%, while the success rate of BIF intubation was higher than that of Macintosh laryngoscopy for patients with tongue edema (84% vs. 76%) [3]. A number of studies reported a success rate of BIF intubation to be 82–100% [6, 11, 12]. In this study, the success rate of intubation with the transillumination-assisted technique was 97.3%, similar to that reported by Sui [13].

In this study, the average intubation time with the BIF was 50.0–52.8 s, which was close to the upper limit of the time range of 23–52 s reported previously [6, 12, 14]. Differences in approach such as left molar approach, para-tongue approach, or modified laryngoscopic structure [15] are possible factors affecting intubation time. Different methods can be used to judge successful intubation, such as the carbon dioxide test or lung auscultation, which may have different durations and lead to different time points, thereby exerting different effects on the results. Some scholars believe that compared to operators who are experienced in conventional laryngoscopy, novices would take shorter time to conduct BIF intubation [2]. When the operators are familiar with the new intubation tools after training, the trainees and experienced operators tended to achieve the same intubation success rate, even in difficult airways. Although the trainees received standard training before the test, they might have different learning abilities, which could lead to differences in intubation time. Thus, although the intubation time did not show any significant difference between the two groups, its clinical significance requires further investigation.

Postoperative laryngeal complications, such as sore throat and hoarseness, are common anesthesia-related complications during tracheal intubation. The frequencies of sore throat and hoarseness were reported to be 11–48% and 18–53%, respectively [16–19]. In this study, no severe intubation-related complications were found, and the frequencies of sore throat and hoarseness in the two groups were consistent with previous studies. In addition, the frequencies of dry throat, sore throat, and hoarseness were found to be significantly higher in the T group than in the R group. The reason for this difference could be a lack of experience in using the intubation technique/instrument. Biro et al. revealed that differences in professional allocation and experience did not significantly affect the incidence and strength of intubation-induced laryngeal complications [20]. On the other hand, Tazeh-Kand et al. showed that the anesthesiologist experience was an independent factor influencing the frequencies of sore throat and hoarseness in male patients after intubation [18]. Recently, Inoue et al. conducted a retrospective propensity score study of 21,606 patients [21] and the frequencies of postoperative laryngeal complications were found not to be significantly different for novices and experienced anesthesiologists. Furthermore, it was believed that laryngeal complications commonly occurred after tracheal intubation, but the incidence was not associated with the experience of the operators [21]. Nevertheless, whether the incidence of complications after BIF intubation is associated with the operators’ experience is still controversial and requires further investigation.

In the transillumination-assisted technique, the operator positions the BIF by finding the bright spot. A direct contact of the tube and laryngoscope tip with the laryngeal tissues leads to intubation-related damage. Although the BIF optical stylet has been proved to be safe and feasible in intubation with transillumination-assisted technique, its light source does not have a penetrability as strong as that of a Trachlight, which may be the reason for more complications in the case of the Bonfils optical stylet [7]. However, in the midline approach technique, the operator adjusts the direction and depth of the laryngoscope by observing through the eyepieces, which minimizes the direct contact of the laryngoscope tip with the vocal cords or epiglottis and other fragile tissues, hence reducing direct damage. The lightwand intubation does not rely on observing through eyepieces, and is not affected by oral secretions, blood, mist, and other factors [22]; this may have more extensive indications compared with the midline approach technique. However, for novices and non-difficult airway, the transillumination-assisted technique may present a higher risk of complications.

This study had the following limitations:

- Premedication: Application of anticholinergic agents before anesthesia can significantly inhibit respiratory tract secretions and create favorable conditions for fiberoptic intubation. However, the common adverse reactions caused by anticholinergic agents (e.g., dry mouth) may lead to postoperative discomfort and other complications. Therefore, anticholinergic agents were not used for medication before anesthesia. However, this may hinder exposing the laryngoscope during intubation. This study was not designed to assess whether anticholinergic agents were associated with increased intubation times.
- Applicability of the instrument: The difficulty in training on the use of the BIF directly leads to poor applicability of this instrument. Currently, many video laryngoscopes are available that are convenient and have a high success rate of intubation. Furthermore, many video laryngoscopes are provided with disposable laryngoscope blades, which can reduce the chance of cross-infection. Nevertheless, since the BIF has its unique scope of application and is difficult to be replaced by other laryngoscopes, it still has potential applications.
- Evaluating intubation safety: The intubation time is unlikely to fully reflect the quality of intubation, while the safety and effectiveness of the operational technique are crucial. In a study by Garcia et al. [23], two layers of pressure-sensitive adhesives were applied to the conventional laryngoscope to measure the intubation force, which was considered an indicator for intubation safety and trainee’s skills. However, this method is not appropriate for evaluating the BIF. In addition, the sensitivity and specificity of methods used to evaluate intubation safety still require a large number of studies. Nevertheless, the transillumination-assisted technique could be more prone to complications, especially for trainees. Therefore, we still recommend the midline approach technique group method.
- Blinding: The two intubation techniques are obviously different in operation process and steps. Therefore, it is impossible to apply blinding to trachea intubation operators, observers, and data collectors. Only the patient could be blinded because they were unconscious during intubation.
- Generalizability: In this study, the patients were relatively young (around 48 years old) and lean (BMI of about 22 kg/m^2^). Therefore, caution should be taken when extrapolating these results to elderly, pediatric, or obese patients.

## Conclusion

For a normal airway, intubation with the transillumination-assisted technique conducted by novices is unlikely to increase the success rate of intubation, but tends to increase the risk of complications. Thus, intubation with the midline approach technique could be safer for novices. The safety and efficacy for novices to manage patients with a difficult airway using the BIF requires further confirmation.

## Additional file

Additional file 1: Figure S1. Linear regression analysis of intubation sequences and intubation time. **Figure S2.** Linear regression analysis of intubation sequences and intubation time. **Figure S3.** Comparison between the two groups suggests that the learning curve of the R group was steeper than that of the T group. (DOCX 234 kb)

## Abbreviations

BIF: Bonfils intubation fiberscope
BIS: Bispectral index
TOF: Train-of-four stimulation
