# Supplementary material for: A comparison of the transillumination-assisted technique versus midline approach technique in novices: a prospective randomized controlled trial about the Bonfils intubation fiberscope
Source: BMC Anesthesiol. 2017 Feb 21;17:31. doi: 10.1186/s12871-017-0322-6 (PMC5320759; doi:10.1186/s12871-017-0322-6)
Supplement: Additional file 1: Figure S1. — Linear regression analysis of intubation sequences and intubation time. Figure S2. Linear regression analysis of intubation sequences and intubation time. Figure S3. Comparison between the two groups suggests that the learning curve of the R group was steeper than that of the T group. (DOCX 234 kb) [file 12871_2017_322_MOESM1_ESM.docx]

**Additional file**


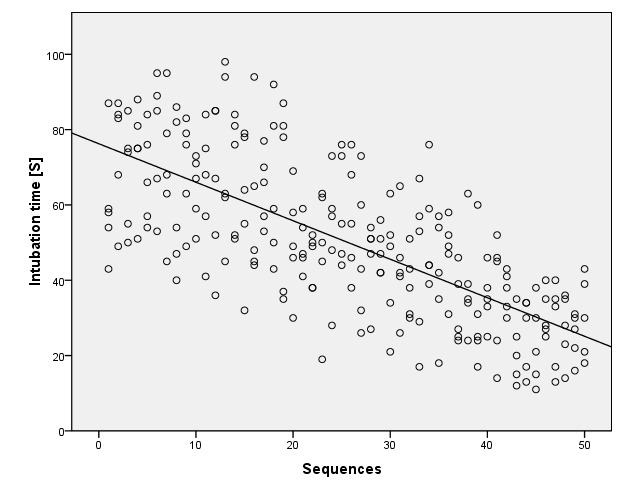


**Figure S1. Linear regression analysis of intubation sequences and intubation time**. The plot displays the degree of linear association between sequences and intubation time of **group T** (y=76.936-0.892x; r=0.717, *P*<0.001). Standard errors of the slope is 0.082.


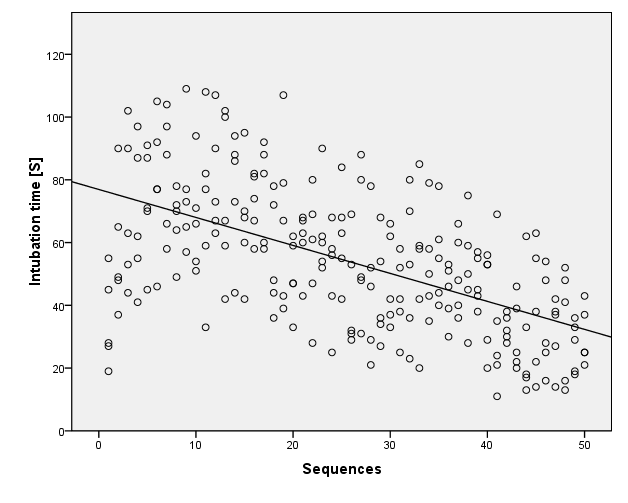


**Figure S2. Linear regression analysis of intubation sequences and intubation time**. The plot displays the degree of linear association between sequences and intubation time of **group R** (y=76.256-1.023x; r=0.569; *P*<0.001). Standard errors of the slope is 0.063.


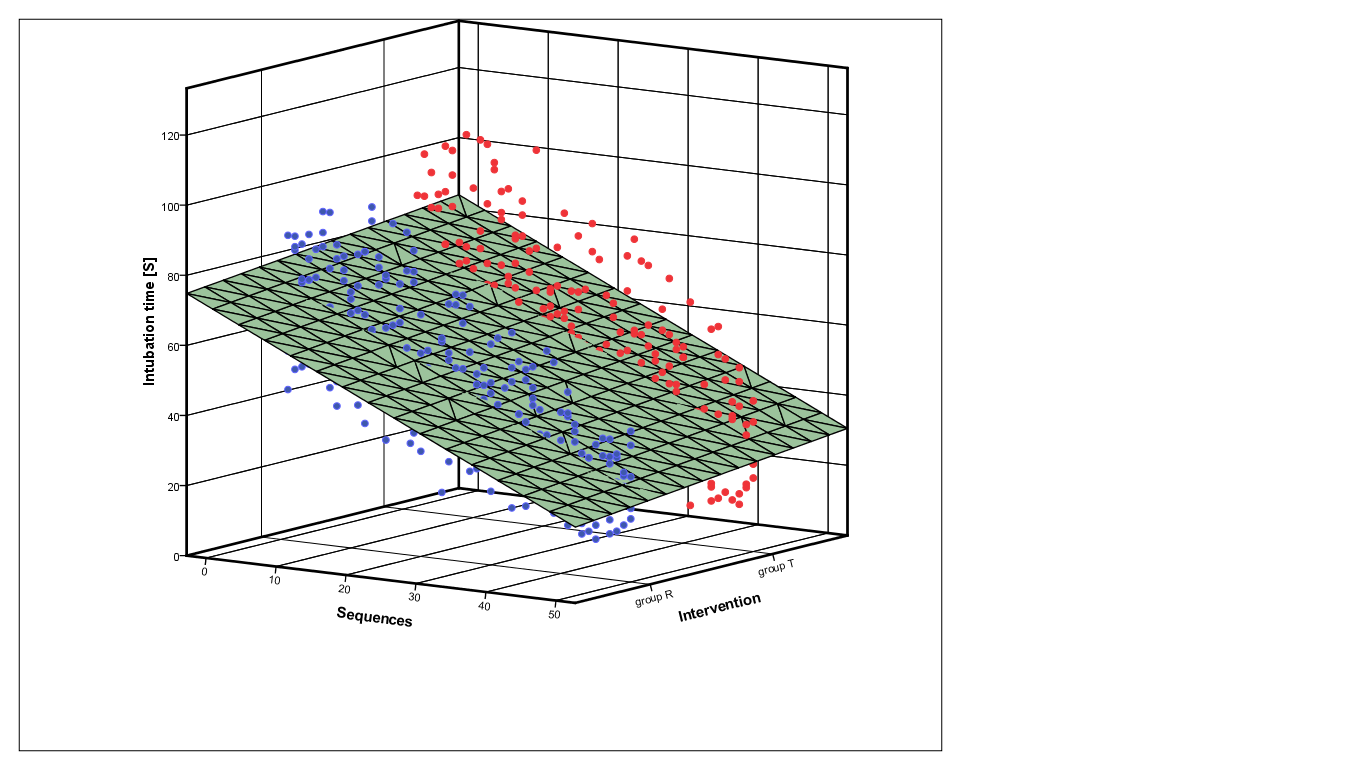


**Figure S3.** Comparison between the two groups suggests that the learning curve of the R group was steeper than that of the T group.
